# Rapid hyperspectral Raman imaging of cells with depthwise separable 3D MultiResU-Net

**DOI:** 10.1016/j.isci.2025.114093

**Published:** 2025-11-18

**Authors:** Weile Zhu, Jianhui Wan, Weina Zhang, Liyun Zhong

**Affiliations:** 1 Institute of Advanced Photonics Technology, School of Information Engineering, Guangdong University of Technology, Guangzhou 510006, China; 2 Key Laboratory of Photonic Technology for Integrated Sensing and Communication, Ministry of Education of China, Guangdong University of Technology, Guangzhou 510006, China; 3 Guangdong Provincial Key Laboratory of Information Photonics Technology, Guangdong University of Technology, Guangzhou 510006, China

**Keywords:** Biocomputational method, Machine learning, Molecular spectra, Neural networks

## Abstract

Hyperspectral Raman imaging (HRI) enables label-free molecular mapping of cells but is constrained by weak Raman scattering signals that require long integration times. We present a depthwise separable three-dimensional MultiResU-Net that performs joint spatial-spectral denoising on full HRI data cubes. By integrating multi-scale feature fusion and depthwise separable convolutions, the network effectively captures spatial-spectral correlations, achieving accurate reconstruction of both spectral features and cellular morphology. Validated on synthetic and experimentally acquired HRI datasets, the proposed method substantially enhances signal quality while preserving structural fidelity. It enables rapid cellular Raman imaging at short integration times without sacrificing spectral integrity, outperforming traditional filters and one-dimensional neural networks. This approach provides an efficient and generalizable computational framework for accelerating HRI, supporting broader applications in high-throughput and dynamic biomedical analysis.

## Highlights

• A depthwise separable 3D MultiResU-Net for denoising hyperspectral Raman images
• Simultaneous processing of spatial and spectral dimensions
• Reduced required integration time from 5 s to 0.5 s per pixel
• On real HeLa cell datasets, the proposed method outperforms traditional methods

## Graphical abstract

**Figure:**
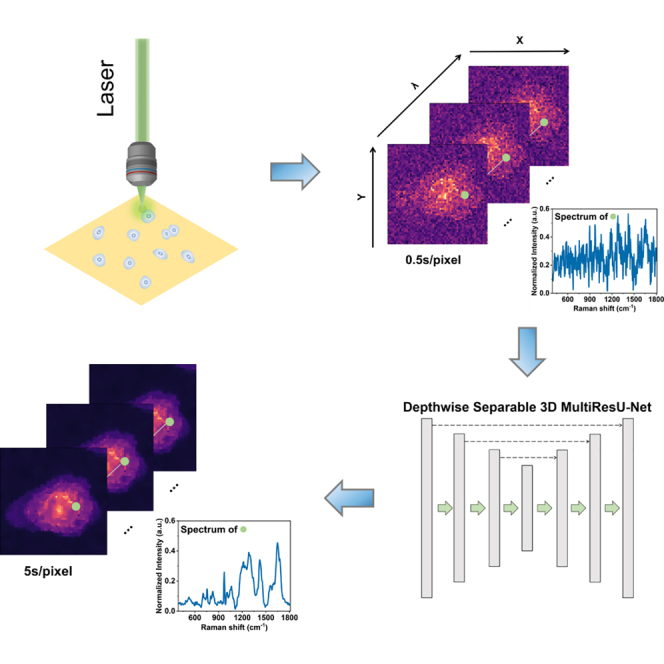

## Introduction

Hyperspectral Raman imaging (HRI), which can simultaneously obtain the chemical information and spatial distribution characteristics of samples, has the advantages of subcellular resolution and is non-destructive and label-free, and has been widely applied in the fields of life sciences,^1^^,^^2^ pharmaceuticals,^3^^,^^4^ and extraterrestrial substance detection.^5^ In particular, cellular HRI enables the visualization of the relative concentrations and spatial distributions of biomolecules such as nucleic acids, lipids, and proteins within cells,^6^ playing a crucial role in the analysis of cellular biochemical processes.^7^ However, Raman signals from cellular samples are typically weak and prone to interference from background noise. High-quality cellular HRI often requires extended acquisition times at the expense of temporal resolution.^8^ In conventional point-scanning Raman imaging, imaging a single cell can take several hours, significantly limiting the study of dynamic processes in living cells.^9^ Therefore, accelerating Raman imaging has become an urgent demand.

To accelerate imaging speed, various strategies have been proposed. One approach focuses on improving the imaging system itself. For instance, multi-focus Raman parallel imaging systems enable simultaneous acquisition of Raman spectra from multiple independent spatial points by employing spatial light modulators and digital micromirror arrays.^10^^,^^11^ Additionally, techniques based on the stimulated Raman principle, such as coherent anti-stokes Raman spectroscopy (CARS) and stimulated Raman spectroscopy (SRS),^12^^,^^13^ have been applied to rapid Raman imaging using pulsed laser systems. However, these system-based acceleration strategies often require complex optical designs and expensive equipment. Another approach to acceleration involves improving signal reconstruction algorithms to improve both the speed and quality of Raman imaging.^14^ A practical and cost-effective alternative is to reduce the exposure time per scan to rapidly acquire low-SNR data, and then apply denoising algorithms to reconstruct high-quality spectra, thereby enabling accelerated Raman imaging.^15^ Traditional denoising algorithms, such as the Savitzky-Golay (S-G) filter,^16^ wavelet denoising,^17^ and principal component analysis (PCA),^18^ are based on certain threshold values for denoising Raman spectra. These methods are prone to misidentifying weak signal peaks as noise or introducing false peaks during noise processing, resulting in spectral distortion and loss of important details, which significantly limits the denoising effect. Deep learning, as a data-driven technique, holds great potential to overcome the limitations mentioned above. By extracting high-dimensional hidden features of data across multiple abstraction layers, deep learning can reveal effective representations of the data,^19^ and has been widely applied in fields such as image classification and segmentation,^20^ natural language processing,^21^ and predictive modeling.^22^ In addition, several recent reviews have highlighted the growing role of artificial intelligence in Raman spectroscopy, particularly in the context of interpretable AI and large-scale models. These works emphasize that AI techniques are becoming central to Raman data analysis and motivate the development of more advanced architectures tailored to biological applications.^23^^,^^24^^,^^25^ With the continuous development of deep learning, deep convolutional neural networks have shown outstanding performance in the preprocessing of Raman spectra.^26^ Ren et al. proposed a rapid Raman imaging method based on convolutional neural networks (CNNs). By widening the slit and using larger scan steps, they obtained low-resolution cellular Raman images, which were then input into a pre-trained CNN to generate high-resolution cellular Raman images. This method achieved a 5-fold increase in speed compared to traditional Raman imaging.^27^ Stevens et al. introduced a deep learning framework named DeepeR to support high-throughput molecular imaging of Raman spectra. The denoising component of this framework took low-SNR spectra collected in 0.1 s and input them into a trained 1D ResU-Net to produce high-SNR spectra that would normally require 1 s of collection time, increasing imaging speed by 10 times.^28^ Wang et al. proposed an MFED network for denoising 1D Raman spectra, incorporating a multi-scale feature fusion module based on the 1D U-Net to enhance the network’s ability to capture features. This method was applied to Raman imaging of cervical cancer cells, achieving a 3-fold increase in speed.^29^ Some recent studies have also explored self-supervised approaches for 1D Raman spectral denoising, but these methods overlook spatial information in hyperspectral imaging.^30^ These approaches have demonstrated the effectiveness of deep learning-based methods in accelerating Raman imaging by denoising low-SNR spectra. However, most of them adopt single-stage denoising architectures that focus either on spectrum-wise denoising or on extracting specific feature peaks from the Raman image.^31^ Such strategies neglect the complex interplay between spatial and spectral information in hyperspectral Raman data, which can lead to the loss of important structural and contextual information during reconstruction, ultimately affecting the accuracy and reliability of the results.

Building on this, this paper proposes an SNR enhancement algorithm that addresses both spectral and spatial dimensions using a depthwise separable 3D MultiResU-Net. This method incorporates depthwise separable 3D convolutions, improving computational efficiency while preserving the correlations between the spectral and spatial dimensions in the reconstructed image. Depthwise separable 3D convolution is a useful lightweight strategy that decomposes a standard 3D convolution kernel into depthwise and pointwise convolutions. This reduces computational costs while maintaining performance similar to that of conventional convolutions. The proposed lightweight network enables efficient and accurate reconstruction with limited training data from cellular Raman images. Specifically, we input low-SNR HeLa cell Raman image volumes, collected at 0.5 s/pixel, into the pre-trained depthwise separable 3D MultiResU-Net and output high-SNR HeLa cell Raman image volumes, which would normally require 5 s/pixel of acquisition time, thus achieving rapid cellular Raman imaging.

## Results

### Data acquisition

Deep learning networks, as data-driven models, require large amounts of data for training. However, in the field of Raman spectroscopy, the number of high-quality public samples is very limited, and no corresponding noisy datasets are available. To better evaluate the denoising performance of the model, we generated low-SNR Raman images by adding noise to high-SNR Raman images from public datasets. We used the synthesized low-SNR images (input) and their corresponding high-SNR images (labels) as data pairs for network training. To further assess the network’s ability to remove real-world noise, we also trained and tested the network with real experimental datasets.

### Synthetic dataset composition

We selected 169 high-SNR hyperspectral Raman images of breast cancer cells from a publicly available dataset (C. C. Horgan, M. Jensen, A. Nagelkerke et al., Anal. Chem. 93(48), 15850–15860, 2021) and used them as labels for generating synthetic noisy training pairs. The image size is 64 × 64×500, with a Raman spectral range of 500–1800 cm^−1^ and a spatial resolution of 0.5 μm. The Raman spectral noise mainly consists of shot noise, dark noise, and readout noise.^32^^,^^33^ Shot noise is inherent to the quantum processes during photon generation and is proportional to the signal intensity, following a Poisson distribution. Readout and dark noise are additive noise sources, independent of signal intensity, and follow a Gaussian distribution. To better reflect the noise distribution in real data, we added a mixed Poisson-Gaussian noise model to the high-SNR breast cancer cell Raman images to generate the noisy dataset. The mathematical expression of the mixed Poisson-Gaussian noise model is as follows:

(Equation 1) Y=αD+E

Where D is the Poisson noise, weighted by the signal intensity. α depends on the sensor and analog gain. E is the Gaussian noise, representing residual noise independent of the signal.

### Real dataset composition

We acquired low-SNR and high-SNR Raman image pairs of HeLa cell samples by performing point scanning with integration times of 0.5 s/pixel and 5 s/pixel, respectively. The image size is 58 × 58×812, with a Raman spectral range of 600–1800 cm^−1^ and a spatial resolution of 1 μm. A total of 144 Raman image pairs were collected in the experiment. After preprocessing, the dataset was divided into 80% for training (115 images), 10% for validation (14 images), and 10% for testing (15 images). To increase the effective size of the dataset and improve the robustness of the network, data augmentation techniques, including image flipping, rotation, and mixing, were applied.

### Network architecture

**Figure 1:**
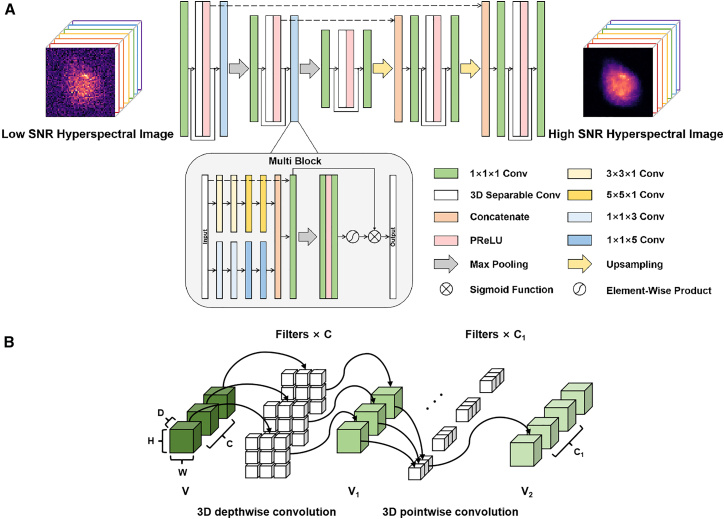
Schematic of the depthwise separable 3D MultiResU-Net (A) Overall framework. (B) Structure of depthwise separable 3D convolution.

The depthwise separable 3D MultiResU-Net is built on the U-Net architecture. U-Net, a special variant of the autoencoder, benefits from its symmetric top-down sampling structure, enabling it to learn from data across multiple feature scales.^34^ The overall framework of the network is shown in Figure 1, consisting mainly of an encoder and a decoder. The encoder part aims to find a lower-dimensional space to represent the input data, while the decoder reconstructs the input data from the compressed representation. The main advantage of the 3D U-Net is its ability to handle 3D volumetric data, which allows it to capture the complex correlations between the spatial and spectral dimensions of spectral data more effectively than traditional 1D methods, achieving more accurate denoising.

However, directly using 3D convolutions requires a very high computational cost, leading to an excessively large number of parameters and making the network difficult to train. To address this, we replace conventional 3D convolutions with depthwise separable 3D convolutions, which have been shown to significantly reduce the number of parameters and floating point operations (FLOPs) while preserving feature extraction capability.^35^ As shown in Figure 1B, depthwise separable 3D convolution decomposes a standard 3D convolution kernel into two separate convolution operations: depthwise convolution, which extracts features from each input channel, and pointwise convolution, which combines all output channels. This approach performs independent calculations for each feature map via depthwise convolution and then uses pointwise convolution to associate the features of each channel, resulting in the final feature map. The introduction of depthwise separable 3D convolution not only reduces computational parameters and simplifies the model to enhance computational efficiency but also accounts for the correlations between the spectral and spatial dimensions. To improve the training effectiveness of the deep network and mitigate the vanishing gradient problem, residual blocks were added to the encoder and decoder. Each residual block consists of a single depthwise separable 3D convolution layer, where each convolution operation includes PReLU activation and batch normalization.

Since cells are biological systems with complex chemical compositions and details at multiple scales, their Raman spectral images are often more subtle and intricate. Therefore, we introduced a multi-scale feature fusion module at each downsampling stage to help the network better capture features at different scales, addressing the issue of excessive information loss during downsampling in U-Net and improving the network’s denoising performance. This multi-scale feature fusion module consists of a multi-scale convolution module and a channel attention module. To reduce the number of computational parameters while accounting for the complex correlations between spatial and spectral dimensions in spectral data, a pseudo-3D convolution was introduced in the multi-scale convolution module. This convolution captures spatial correlations by applying a 3 × 3×1 convolution in the spatial domain and maintains spectral continuity between adjacent frames by applying a 1 × 1×3 convolution in the spectral domain, with the extracted features from both convolutions concatenated. Specifically, the multi-scale convolution module includes two 3 × 3×1 standard convolutions and two 5 × 5×1 standard convolutions in the spatial domain, as well as one 1 × 1×3 standard convolution, one 1 × 1×5 standard convolution, one 1 × 1×5 dilated convolution, and one 1 × 1×7 dilated convolution in the spectral domain, with dilation rates of 5 and 7, respectively. The features extracted from both the spatial and spectral domains are then fused and concatenated. By using convolution layers with different kernel sizes, the network can detect features at different scales, and the introduction of dilated convolutions increases the network’s receptive field in the spectral domain, thus better linking contextual features.

The model was trained for 100 epochs with a batch size of 2, using the Adam optimizer to optimize the gradients. The learning rate was initially set to 0.001 and reduced to 0.0001 at the 60th epoch. The loss function was defined as a weighted combination of L1 and MSE terms, with weights of 0.1 and 0.9, respectively. The network was trained separately on two datasets: one with synthetic noisy-clean Raman image pairs and the other with experimentally acquired Raman image pairs, both starting from scratch. The sensitivity of hyperparameters such as learning rate and batch size was further evaluated (Tables S1 and S2).

### Denoising results on synthetic noise data

**Figure 2:**
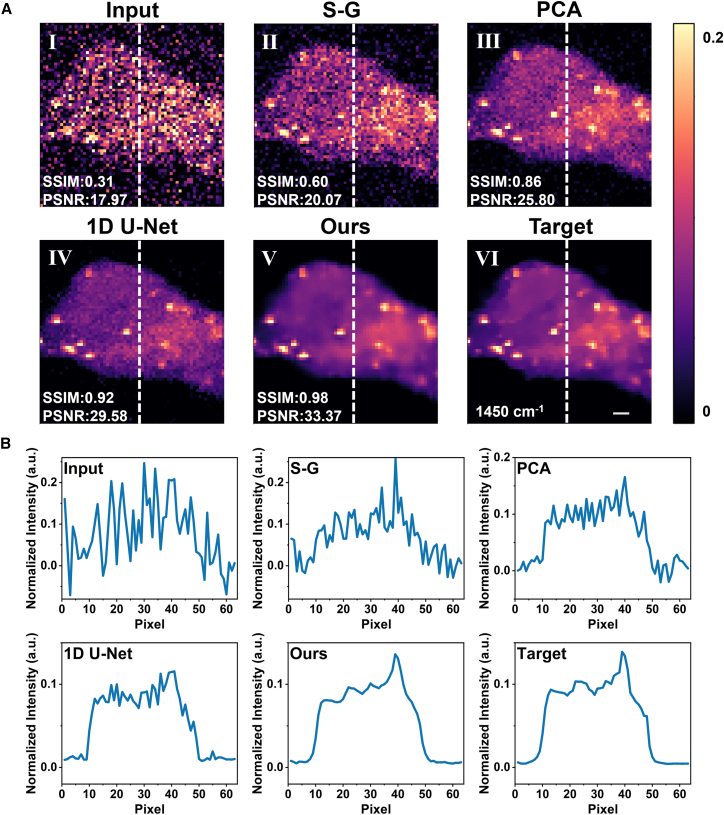
Spatial reconstruction results of breast cancer cell Raman images with mixed noise (A) Intensity maps at 1450 cm^−1^: (I) noisy input, (II) S-G filter, (III) PCA, (IV) 1D U-Net, (V) proposed method, and (VI) high-SNR ground truth. (B) Intensity profiles along the white dashed lines in (a). Scale bars = 2.5 μm.

We trained and tested our model on synthetic noise data created by adding mixed Poisson-Gaussian noise to breast cancer cell data. The denoising results of our proposed depthwise separable 3D MultiResU-Net were compared with those of the S-G filter, PCA, and 1D U-Net. Figure 2 shows the results on test samples that were not used in the training of the network. In Figure 2A, we selected the peak intensity map corresponding to the wavelength of 1450 cm^−1^ (a representative Raman peak). For the S-G filter, PCA, and 1D U-Net, denoising was first performed on individual spectra in the 3D dataset, and the results were then reassembled into the denoised 3D dataset. In contrast, our proposed method directly processes the entire 3D dataset as input. From the intensity maps in Figure 2A II-III, the S-G filter provides only limited noise suppression, while PCA achieves noticeably better denoising but still leaves residual graininess and incomplete recovery of cellular morphology. The 1D U-Net (Figure 2A IV) further improves image clarity and suppresses noise more effectively than PCA; however, faint background speckles persist, and some fine structural details are not fully restored. In comparison, our proposed method (Figure 2A V) removes most of the residual noise while better preserving cellular morphological details, leading to a significant improvement in image quality. Figure 2B presents the intensity curves corresponding to the white dashed lines in Figure 2A. Compared with other methods, the intensity curve of the reconstruction obtained using our proposed method is closest to the ground truth, further validating its effectiveness. The quantitative evaluation of PCA denoising performance with different numbers of retained components is provided in Table S3. For completeness, additional spatial reconstruction results at the 1660 cm^−1^ peak are provided in Figure S1.

**Figure 3:**
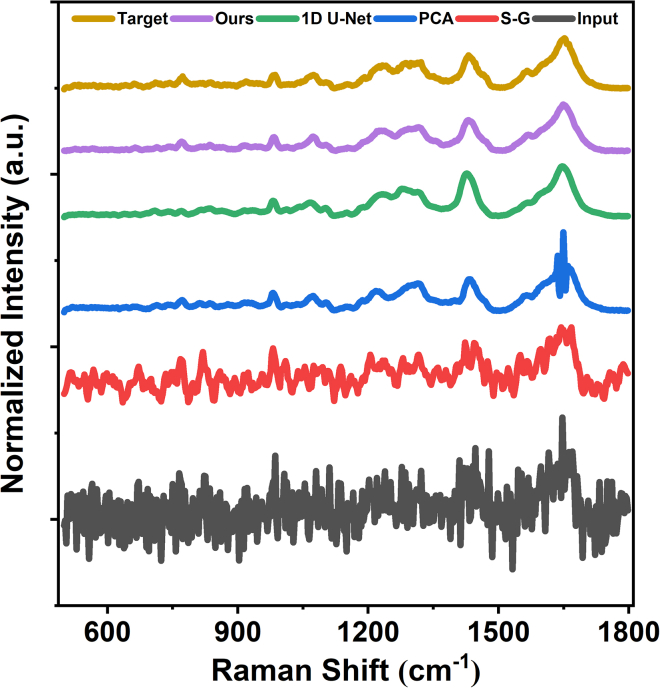
Raman spectra from the same pixel reconstructed by different denoising methods All spectra are plotted on the same Raman-shift axis and vertically offset for clarity.

To assess performance in the spectral domain, we extracted the spectra from the same pixel across all reconstructions for a side-by-side comparison. Figure 3 shows the noisy input, the spectra denoised by the S-G filter, PCA, and 1D U-Net, the reconstruction obtained by the proposed method, and the high-SNR reference. For clarity, all spectra are plotted on the same Raman-shift axis and vertically offset. The S-G filter suppresses part of the high-frequency noise but leaves noticeable fluctuations. PCA provides substantially stronger noise suppression than the S-G filter; however, residual fluctuations remain in the spectra, and slight distortions in peak shapes and relative intensities can be observed. The reconstruction from the 1D U-Net appears close to the target overall, but it fails to accurately reproduce the characteristic DNA peak at 795 cm^−1^, indicating limited reliability in capturing critical biochemical information. In contrast, the proposed method most faithfully preserves peak positions and relative intensities while effectively reducing background fluctuations, yielding the closest match to the high-SNR reference.

To quantitatively evaluate the similarity between the reconstructed results and the original high-SNR data, we assessed both the spatial and spectral dimensions using appropriate metrics. For the spatial dimension, PSNR and SSIM were employed. A higher PSNR indicates better image quality, while SSIM values range from 0 to 1, with higher values representing greater structural similarity to the reference image. For the spectral dimension, we used MSE and SNR. Lower MSE indicates better spectral alignment, and higher SNR corresponds to improved spectral quality. The formulas for these metrics are provided in the Materials and Methods section. Quantitative comparisons of different reconstruction methods were performed in both dimensions, and the average results across the entire test set are summarized in Table 1 (spatial metrics) and Table 2 (spectral metrics).

**Table 1:** Quantitative comparison of spatial reconstruction quality on the synthetic dataset

	Input	S-G	PCA	1D U-Net	Ours
SSIM	0.32	0.60	0.93	0.98	0.99
PSNR	23.79	24.91	38.48	44.69	48.98

Values represent the mean over *n* = 8 independent samples from the synthetic validation dataset.

**Table 2:** Quantitative comparison of spectral reconstruction quality on the synthetic dataset

	Input	S-G	PCA	1D U-Net	Ours
MSE	3.20 × 10^−3^	8.47 × 10^−4^	1.46 × 10^−4^	1.06 × 10^−4^	1.41 × 10^−5^
SNR	−4.77	1.04	9.24	14.99	21.53

Values represent the mean over *n* = 8 independent samples from the synthetic validation dataset.

As shown in Tables 1 and 2, the proposed method outperforms all comparison approaches in both the spatial and spectral dimensions. Specifically, it achieved an SSIM of 0.99 and a PSNR of 48.98 dB for the reconstructed image, and an MSE of 1.41 × 10^−5^ and an SNR of 21.53 dB for the reconstructed spectrum. These results demonstrate that the depthwise separable 3D MultiResU-Net effectively captures both spatial structures and spectral features of Raman images, successfully modeling the complex spatial-spectral correlations and enhancing its ability to distinguish true Raman signals from high background noise.

### Experimental data denoising results

**Figure 4:**
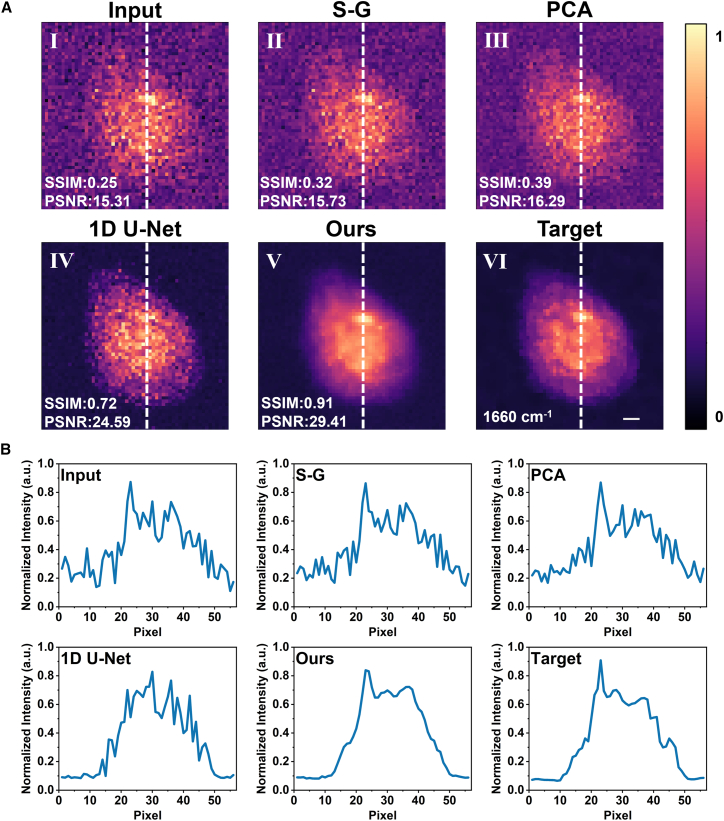
Spatial reconstruction results of HeLa cell Raman images (A) Intensity maps at 1660 cm^−1^: (I) noisy input (0.5 s/pixel), (II) S-G filter, (III) PCA, (IV) 1D U-Net, (V) proposed method, and (VI) high-SNR ground truth (5 s/pixel). (B) Intensity profiles along the white dashed lines in (A). Scale bars = 5 μm.

In real sample experiments, we trained and tested the model on HeLa cell data collected using short (0.5 s/pixel) and long (5 s/pixel) integration times. Figure 4 shows the results on test samples that were not used for training. For comparison, we also present the results of S-G filtering and PCA. Figure 4A I shows the 2D intensity map at 1660 cm^−1^ extracted from the raw low-SNR Raman data cube acquired with a 0.5 s/pixel integration time. Figure 4A II and 4a III display the corresponding intensity maps after S-G filtering and PCA, respectively; Figure 4A IV shows the result of 1D U-Net; and Figure 4A V presents the reconstruction obtained by the proposed depthwise separable 3D MultiResU-Net. It is evident that both S-G filtering and PCA still retain considerable noise, which causes the essential cellular information to be obscured. The reconstruction using a 1D U-Net restores cellular morphology relatively well, but the cell-background boundaries remain blurred, likely due to the failure to account for the spatial dimension. In contrast, the depthwise separable 3D MultiResU-Net effectively addresses this issue and aligns closely with the target reconstruction. Figure 4B presents the intensity curves along the white dashed line in Figure 4A. Compared to the other methods, the intensity curve of the reconstructed image from the proposed method aligns more closely with the true value, further demonstrating that the proposed method is highly effective in restoring detailed image information.

**Figure 5:**
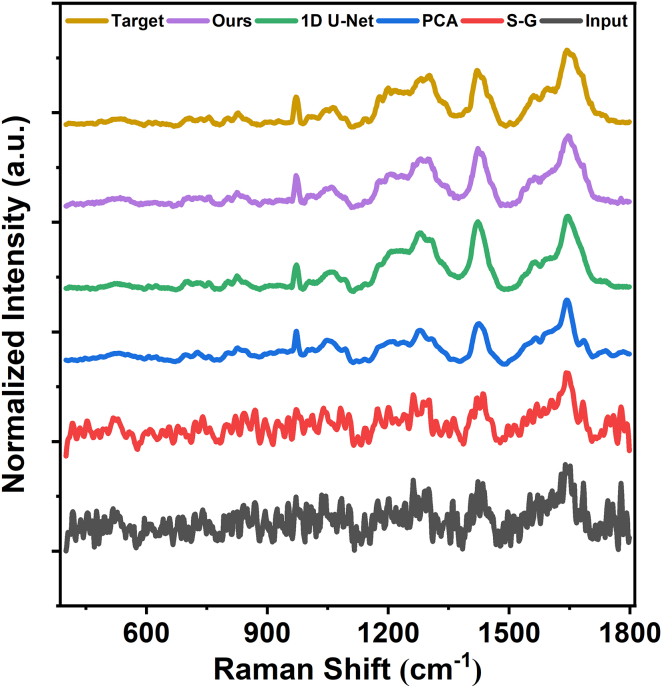
Raman spectra of HeLa cells reconstructed by different denoising methods All spectra are plotted on the same Raman-shift axis and vertically offset for clarity.

Similarly, we evaluated the spectral reconstruction performance on experimentally acquired HeLa cell data. Figure 5 shows the low-SNR input spectrum (0.5 s/pixel), the spectra reconstructed using the S-G filter, PCA, 1D U-Net, and the proposed depthwise separable 3D MultiResU-Net, together with the high-SNR reference spectrum (5 s/pixel). For clarity, all spectra are plotted on the same Raman-shift axis and vertically offset. The S-G filter reduces some fluctuations but leaves substantial noise. PCA provides stronger noise suppression than the S-G filter, but residual variations and distortions in relative peak intensities remain. The 1D U-Net reconstruction shows closer agreement with the reference, yet the intensity of the 1660 cm^−1^ characteristic peak is not accurately recovered. In contrast, the proposed method achieves the best overall performance, effectively suppressing noise while maintaining strong consistency in both peak positions and intensities with the high-SNR reference.

We conducted a quantitative evaluation of different reconstruction methods in the spatial dimension using PSNR and SSIM, as shown in Table 3. As summarized in Table 3, the proposed depthwise separable 3D MultiResU-Net consistently outperforms traditional methods such as S-G filtering, PCA, and the 1D U-Net. With a superior PSNR of 34.11 dB, the method effectively suppresses noise, leading to clearer and higher-quality images. Additionally, its significantly higher SSIM of 0.91 demonstrates its ability to preserve fine details and structural features. In contrast to the 1D U-Net, which only processes individual spectral dimensions sequentially, the proposed method leverages the power of depthwise separable 3D convolutions to capture spatial correlations comprehensively, resulting in more accurate reconstructions.

**Table 3:** Quantitative comparison of spatial reconstruction quality on the experimental dataset

	Input	S-G	PCA	1D U-Net	Ours
SSIM	0.13	0.17	0.44	0.80	0.91
PSNR	15.73	16.02	16.54	31.23	34.11

Values represent the mean over *n* = 15 independent HeLa cell samples from the experimental validation dataset.

To comprehensively evaluate the performance of different reconstruction methods in the spectral dimension, we used two key metrics: MSE and SNR. The quantitative comparison presented in Table 4 shows that the proposed method achieves the best performance, with an MSE of 4.66 × 10-4 and an SNR of 15.67 dB, outperforming the 1D U-Net. This indicates that the proposed method excels in both reconstruction accuracy and spectral quality, effectively preserving the signal while minimizing noise. To further evaluate the contribution of the multi-scale feature fusion module, we conducted an ablation experiment on the HeLa cell dataset. The quantitative results (Table S4) demonstrate that incorporating this module consistently improves SSIM, PSNR, MSE, and SNR, confirming its effectiveness in enhancing reconstruction quality.

**Table 4:** Quantitative comparison of spectral reconstruction quality on the experimental dataset

	Input	S-G	PCA	1D U-Net	Ours
MSE	2.79 × 10^−2^	2.61 × 10^−2^	2.37 × 10^−2^	8.06 × 10^−4^	4.66 × 10^−4^
SNR	−5.26	−4.96	−4.51	14.36	15.67

Values represent the mean over *n* = 15 independent HeLa cell samples from the experimental validation dataset.

Furthermore, to assess the generalization capability of the proposed model, we tested it on apoptotic HeLa cells not included in the training set. The reconstruction results (Figure S2) demonstrate that the model maintains high denoising performance under different cellular conditions, indicating good generalization potential.

## Discussion

The proposed depthwise separable 3D MultiResU-Net enables volumetric learning across spatial-spectral domains for hyperspectral Raman imaging, achieving efficient denoising while preserving fine molecular details. Compared with PCA and 1D U-Net, the model demonstrates superior quantitative performance in both spatial and spectral fidelity across synthetic and experimental datasets, indicating improved reconstruction accuracy and robustness.

This performance improvement stems from two major architectural designs. First, the depthwise separable 3D convolution effectively reduces redundant parameters while maintaining the network’s ability to capture cross-dimensional features. Second, the multi-scale feature fusion module enhances sensitivity to subtle Raman peaks and suppresses random noise through hierarchical feature extraction. These improvements allow the network to better reconstruct both cellular morphology and spectral information simultaneously.

Unlike traditional one-dimensional Raman denoising models, which process spectra independently, the proposed framework performs 3D contextual learning over the entire data cube, allowing simultaneous recovery of morphological and spectral information. This approach achieves practical one-order-of-magnitude faster imaging (0.5 s/pixel) without compromising spectral precision, paving the way for real-time, label-free cellular Raman imaging and broader biomedical applications.

Future work may focus on integrating physical priors and self-supervised learning strategies to enhance model interpretability, robustness, and adaptability under varying experimental conditions.

### Limitations of the study

Despite the promising results, this study has several limitations. First, the datasets used for training and testing are relatively small, which may limit the generalization ability of the proposed model. Second, slight over-smoothing is observed in the spatial cross-sectional images corresponding to the 1450 cm^−1^ characteristic peak, particularly under low SNR conditions. Third, the current validation is limited to breast cancer and HeLa cells, and further studies on diverse cell types and experimental conditions are needed to confirm the robustness of the method. In particular, the lack of validation on additional cell lines remains a limitation that should be addressed in future work. Moreover, the comparative experiments were restricted to the widely used 1D U-Net baseline; incorporating more advanced spectral and image denoising algorithms could provide a more comprehensive evaluation.

## Resource availability

### Lead contact

• Requests for further information and resources should be directed to and will be fulfilled by the Lead contact, Liyun Zhong (zhongly@gdut.edu.cn).

### Materials availability

• This study did not generate new unique reagents.

### Data and code availability

• The breast cancer Raman dataset used for synthetic noise experiments is publicly available at https://github.com/conor-horgan/(Horgan et al., Analytical Chemistry 93(48), 15850–15860, 2021). The Raman datasets of HeLa cells reported in this paper will be shared by the lead contact upon request. All other data reported in this paper will be available from the lead contact upon request.
• All original code has been deposited at GitHub and is publicly available at https://github.com/MRWEILE/Depthwise-Separable-3D-MultiResU-Net as of the date of publication.
• Any additional information required to reanalyze the data reported in this paper is available from the lead contact upon request.

## Acknowledgments

This work is supported by the 10.13039/501100001809National Nature Science Foundation of China (Grant No. 62175041, 62127816, 61875059 and 12004444); the Guangdong Introducing Innovative and Entrepreneurial Teams of the “The Pearl River Talent Recruitment Program” (Grant No. 2019ZT08X340); the Guangdong Provincial Key Laboratory of Information Photonics Technology (Grant No. 2020B121201011); the 10.13039/501100021171Guangdong Basic and Applied Basic Research Foundation (No. 2024A1515011728); and the Guangzhou Basic and Applied Basic Research Foundation (No. 2023A04J2043).

## Author contributions

Conceptualization, W.Z. and L.Z.; methodology, W.Z. and J.W.; investigation, W.Z. and W.Zh.; writing – original draft, W.Z.; writing – review and editing, W.Z., W.Zh., and L.Z.; funding acquisition, L.Z. and W.Zh.; resources, W.Zh. and L.Z.; supervision, L.Z. and J.W.

## Declaration of interests

The authors declare no competing interests.

## STAR★Methods

### Key resources table

**Table:** 

REAGENT or RESOURCE	SOURCE	IDENTIFIER
**Chemicals, peptides, and recombinant proteins**		

4-Mercaptopyridine	Beijing Putian Tongchuang Co., Ltd.	CAS# 1129-06-2
DMEM (1×)	Gibco, Thermo Fisher Scientific	Cat# 11995065
Fetal bovine serum (FBS)	Gibco, Thermo Fisher Scientific	Cat# 10099141
Penicillin-streptomycin (1×)	Gibco, Thermo Fisher Scientific	Cat# 15140122
PBS buffer (pH 7.4, 1×)	Gibco, Thermo Fisher Scientific	Cat# 10010023
Milli-Q ultrapure water	Millipore	N/A

**Deposited data**		

Breast cancer Raman dataset	Horgan et al., Anal. Chem. (2021)	https://github.com/conor-horgan/
HeLa Raman datasets	This paper	Available from Lead contact upon request

**Experimental models: cell lines**		

HeLa cells	Cell Bank of the Chinese Academy of Sciences, Shanghai, China	Cat# SCSP-504

**Software and algorithms**		

WiRE software (v5.6)	Renishaw	https://www.renishaw.com
PyTorch (v2.3.1)	PyTorch Foundation	https://pytorch.org
NVIDIA CUDA (v12.6)	NVIDIA	https://developer.nvidia.com/cuda-zone
Depthwise Separable 3D MultiResU-Net	This paper	https://github.com/MRWEILE/Depthwise-Separable-3D-MultiResU-Net

### Experimental model and study participant details

#### Cells and reagents

HeLa cells (SCSP-504; Cell Bank of the Chinese Academy of Sciences, Shanghai, China) were used in this study. HeLa is an immortalized human cervical cancer cell line derived from a female donor. After thawing, cells were cultured in DMEM (1×) supplemented with 10% fetal bovine serum (FBS) and 1% penicillin–streptomycin at 37°C in a humidified incubator with 5% CO_2_, with medium changes every 2–3 days and passaging as needed; new culture flasks were used every 5–6 passages. Prior to Raman imaging, cells were fixed on gold-coated substrates. According to the provider’s quality control documentation, this cell line is free of mycoplasma, bacterial, and fungal contamination and has been authenticated by short tandem repeat (STR) profiling; no additional authentication was performed by the authors. 4-Mercaptopyridine was obtained from Beijing Putian Tongchuang Co., Ltd. DMEM, PBS (pH 7.4, 1×), penicillin–streptomycin (0.25% Trypsin-EDTA, 1×), and FBS were purchased from Gibco (USA), and ultrapure water from a Milli-Q Millipore system was used for all experiments. All chemicals were of analytical grade and used without further purification. No animals or human participants were directly involved in this study. HeLa cells are derived from a female donor; sex was not considered as an experimental variable in this study, and all results should be interpreted with this limitation in mind.

### Method details

#### Equipment and characterization

Raman spectra were collected using a Renishaw inVia confocal Raman microscope. The Raman excitation wavelength was 532 nm, and the excitation power was set to 12.5 mW. A 50X objective lens (Leica), NA=0.5, was used to scan the HeLa cell Raman images. The computing system included an NVIDIA RTX 4090 GPU and an Intel Core i9-13900KS processor. The environment was configured with torch 2.3.1, NVIDIA CUDA 12.6, Cudnn 9.5.1, and the Windows 10 operating system.

#### Data preprocessing

The WiRE software (version 5.6) was used for preprocessing and analysis of the collected Raman images, including cosmic ray removal, baseline correction, and noise smoothing. The intensity of each Raman spectrum image depends on sample characteristics, the acquisition environment, and the Raman spectrometer. Therefore, to eliminate intensity inhomogeneity between spectral images, the Raman spectral image data was normalized using the min - max scaling method.The min - max normalization formula for Raman spectral images is as follows:

(Equation 2) $${X_{i}}^{\prime }=\frac{X_{i}-X_{\min}}{X_{\max}-X_{\min}}$$

Where Xmax and Xmin represent the maximum and minimum intensity values in the hyperspectral image, respectively. The normalized hyperspectral image size is H×W×B, where H, W, and B represent the height, width, and spectral channels of the Raman spectral image, respectively.

### Quantification and statistical analysis

All quantitative evaluations focused on comparing reconstruction performance between the proposed depthwise separable 3D MultiResU-Net and baseline methods in both spatial and spectral domains. Four metrics were used: structural similarity index (SSIM) and peak signal-to-noise ratio (PSNR) for image quality, and mean squared error (MSE) and signal-to-noise ratio (SNR) for spectral fidelity. All metrics were implemented using custom Python scripts based on PyTorch and NumPy.

For the synthetic dataset, values reported in Tables 1 and 2 correspond to the mean performance over n = 8 independent samples from the synthetic validation dataset for each method. For the experimental HeLa dataset, values in Tables 3 and 4 correspond to the mean performance over n = 15 independent samples from the experimental validation dataset for each method. In all cases, n denotes the number of independent hyperspectral Raman image cubes (samples), and the center is defined as the arithmetic mean. No error bars are plotted in the main figures; pooled results are presented as descriptive means, as indicated in the figure legends and table footnotes.

No formal hypothesis testing or p value–based significance analysis was performed, as the primary objective was to provide deterministic, reproducible quantitative comparisons of denoising performance rather than to test biological hypotheses. Accordingly, no randomization, stratification, or prospective sample size estimation procedures were applied beyond the predefined training/validation/testing splits of the datasets. All samples that passed basic quality control were included, and no data points were excluded based on outcome.

#### Evaluation metrics

The SSIM between two images, x and y, is defined as:

(Equation 3) $$\text{SSIM}\left(x,y\right)=\frac{\left(2\mu_{x}\mu_{y}+C_{1}\right)\left(2\sigma_{\text{xy}}+C_{2}\right)}{\left({\mu_{x}}^{2}+{\mu_{y}}^{2}+C_{1}\right)\left({\sigma_{x}}^{2}+{\sigma_{y}}^{2}+C_{2}\right)}$$

where μ_x_ and μ_y_are the mean values of x and y, ${\sigma_{x}}^{2}$ and ${\sigma_{y}}^{2}$ are the standard deviations of x and y, and σ_xy_ is the covariance between x and y. C_1_ and C_2_ are constants to stabilize the division with weak denominators. For hyperspectral Raman images, SSIM is calculated independently for each channel and then averaged.

The PSNR of image y relative to the reference image x is defined as:

(Equation 4) $$\text{PSNR}\left(x,y\right)=10\times \log_{10}\frac{R}{{\left(y-x\right)}^{2}}$$

where R is the maximum possible pixel value in x, and MSE is the Mean Squared Error between x and y.

The MSE between two spectra a and b is given by:

(Equation 5) $$\text{MSE}=\frac{1}{m}\sum_{i=1}^{m}{\left[b\left(i\right)-a\left(i\right)\right]}^{2}$$

where m is the number of spectral data points, and a(i) and b(i) are the values of the spectra at the i-th point.

The SNR for spectra a and b is defined as:

(Equation 6) $$\text{SNR}=10\times \log_{10}\frac{a^{2}}{{\left(b-a\right)}^{2}}$$

where the numerator represents the signal power, and the denominator represents the noise power, calculated as the squared difference between the reconstructed spectrum and the reference spectrum.

These metrics allow for a comprehensive evaluation of the reconstruction quality, both in terms of image similarity and spectral fidelity, and provide a basis for comparison with other denoising methods.
