# Supplementary material for: Rapid hyperspectral Raman imaging of cells with depthwise separable 3D MultiResU-Net
Source: iScience. 2025 Nov 18;28(12):114093. doi: 10.1016/j.isci.2025.114093 (PMC12702228; doi:10.1016/j.isci.2025.114093)
Supplement: Document S1. Figures S1 and S2 and Tables S1–S4 [file mmc1.pdf]

iScience, Volume *28*

Supplemental information

Rapid hyperspectral Raman imaging of cells with depthwise separable 3D MultiResU-Net

Weile Zhu, Jianhui Wan, Weina Zhang, and Liyun Zhong

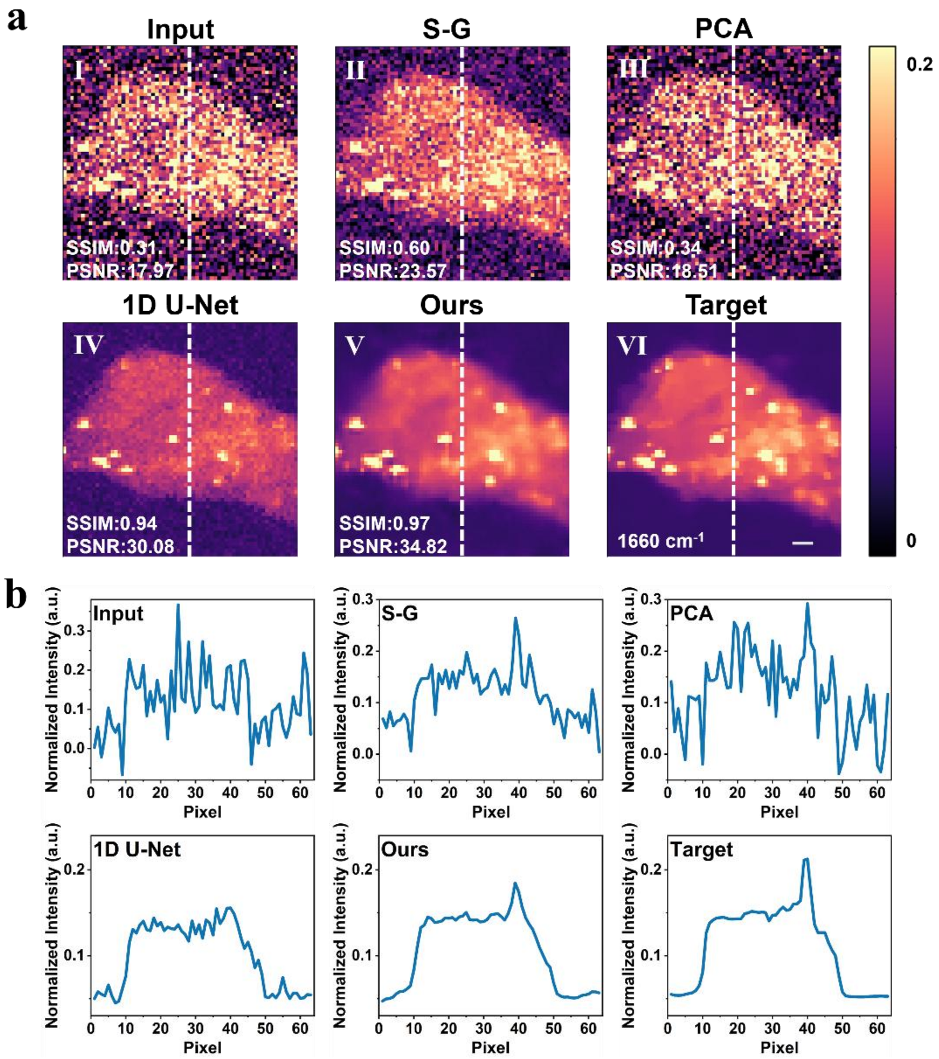

Figure S1. Spatial reconstruction results of breast cancer cell Raman images at the 1660 cm^{-1} peak under mixed noise conditions.

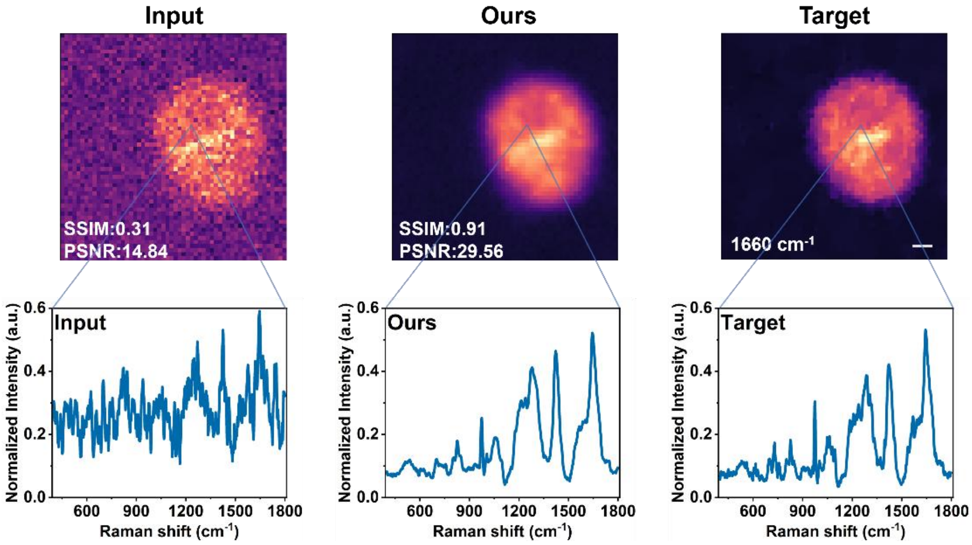

Figure S2. Generalization test of the denoising network on paclitaxel-induced apoptotic HeLa cells.

Table S1. Hyperparameter Sensitivity Analysis of the 3D Network Performance

batch size	lr	n_conv	SSIM	PSNR	MSE	SNR
1	1e-3	1	0.91	32.78	6.07×10^{-4}	13.97
2	1e-3	2	0.90	34.11	4.81×10^{-4}	15.56
2	1e-3	1	**0.91**	**34.11**	$\mathbf{4.66\times10^{-4}}$	**15.67**
2	5e-3	1	0.91	33.55	5.63×10^{-4}	14.95
2	5e-4	1	0.90	33.58	5.14×10^{-4}	14.84

Table S2. Hyperparameter sensitivity analysis of the 1D U-Net

Batch size	lr	SSIM	PSNR	MSE	SNR
128	5e-5	0.79	**31.44**	8.96×10^{-4}	14.37
256	5e-5	0.82	30.63	9.11×10^{-4}	14.03
512	5e-5	0.80	31.23	$\mathbf{8.06\times10^{-4}}$	14.36
512	5e-4	0.76	30.34	1.01×10^{-3}	**14.52**
512	1e-5	**0.83**	30.03	1.10×10^{-3}	12.48

Table S3. Performance of PCA denoising under varying numbers of components

Number of Principal Components	SSIM	PSNR	MSE	SNR
10	0.94	39.96	1.05×10^{-4}	10.90
12	0.94	39.19	1.21×10^{-4}	10.17
15	0.93	38.48	1.46×10^{-4}	9.24
18	0.93	37.79	1.70×10^{-4}	8.48
20	0.92	37.40	1.86×10^{-4}	8.05

Table S4. Performance comparison with and without the feature fusion module

	SSIM	PSNR	MSE	SNR
with	**0.91**	**34.11**	$\mathbf{4.66\times10^{-4}}$	**15.67**
without	0.90	32.88	6.64×10^{-4}	14.39
